# Genome Characterization of *Lactiplantibacillus plantarum* Strain UTNGt2 Originated from *Theobroma grandiflorum* (White Cacao) of Ecuadorian Amazon: Antimicrobial Peptides from Safety to Potential Applications

**DOI:** 10.3390/antibiotics10040383

**Published:** 2021-04-03

**Authors:** Gabriela N. Tenea, Clara Ortega

**Affiliations:** Biofood and Nutraceutics Research and Development Group, Faculty of Engineering in Agricultural and Environmental Sciences, Technical University of the North, Av. 17 de Julio s-21 y José María Córdova, Barrio El Olivo, 100150 Ibarra, Ecuador; cgortega@utn.edu.ec

**Keywords:** genome analysis, *Lactiplantibacillus plantarum*, antimicrobial peptides, food safety, RiPP-like peptides, plantaricin

## Abstract

The genome characterization of the *Lactiplantibacillus plantarum* strain UTNGt2, isolated from wild copoazu or white cacao (*Theobroma grandiflorum*), is described. A total of 31 contigs is assembled with a total length of 3,264,448 bases, with all contigs matching the core genome of different groups in the database. The genome size is 3,540,752 bases with GC content of 44.53% and the genome repeat sequences constitute around 457,386 bases of the assembly. The UTNGt2 matches the *Lactiplantibacillus plantarum* genome with 99% identity. The genome contains 3115 genes, 3052 protein-coding genes, assigned with the EggNOG database. On the basis of the results, 745 proteins are classified with an unknown function, from which 128 proteins have no match in the BLASTN database. It also contains 57 tRNAs, 5 copies of 5S rRNA, and 1 copy of tmRNA. Based on gene prediction and annotation results, 9.4% of proteins are involved in carbohydrate transport and metabolism and 8.46% in transcription, 2.36% are responsible for defense mechanisms, 0.5% are responsible for the biosynthesis of secondary metabolites, transport, and catabolism, while 25.11% have an unknown function. The genome revealed the presence of genes involved in riboflavin and folate production, the presence of CRISPR/Cas genes, phage sequences, the absence of acquired antibiotics resistance genes, virulence, and pathogenic factors, suggesting that UTNGt2 is a safe strain. Its highly antimicrobial capacity is related to the presence of two bacteriocin clusters (class IIc) of the sactipeptide class (contig 4) and plantaricin E class (contig 22), as detected by the BAGEL 4 webserver. Several RiPP-like peptides (non-bactericidal ribosomally produced and post-translationally modified peptides), polyketides (PKs), and terpenes were predicted. Whole-genome sequencing analysis revealed that the UTNGt2 strain has diverse bacteriocins with a high inhibitory capacity, thus it is a bacteriocinogenic strain. Considering the safety profile, UTNGt2 is a nonpathogenic, nonvirulent strain with valuable biotechnological traits and can be further exploited for its probiotic and antimicrobial potential in the food industry or as a potential producer strain of antimicrobial peptides as an alternative to conventional antibiotics.

## 1. Introduction

Lactic acid bacteria (LAB) are a group of microorganisms ubiquitously distributed in nature [1,2]. Due to their probiotic and antimicrobial characteristics, several LAB species are incorporated in different foods conferring health benefits to consumers, as well they contribute to food safety by inhibiting the proliferation of undesirable spoilage or pathogenic microorganisms [3,4,5,6,7,8].

*Lactobacillus* species are considered as natural food ingredients, for their use in human nutrition as a starter culture and are recognized as GRAS (Generally Recognized as Safe or Qualified Presumption of Safety) by the FDA Food and Drug Administration and EFSA (European Food Safety Authority) [9,10]. According to EFSA guidelines, the newly isolated strains intended to be used in foods should be characterized in terms of taxonomic identification, pathogenicity state, production of antimicrobials, genome sequencing, and characterization being considered straightforward diagnostic tools [11].

Various *Lactobacillus* species and strains draw interest for the development of new antimicrobials, functional foods, and the next generation of probiotics. Bacteriocins, produced by lactobacilli are the most promising antimicrobial candidates [11]. Besides, some strains harbor naturally CRISPR (Clustered Regularly Interspaced Short Palindromic Repeats) arrays and Cas associated proteins, which grant the capacity to be converted into a tool for genome editing [12]. Most LAB species used as probiotics or antimicrobial producers are of human or animal origin, and recently, several scientific documents indicated the probiotic potential of LAB species isolated from unconventional sources such as fruits, vegetables, juices, and grain products [8,13,14]. Among Lactobacilli, *Lactobacillus plantarum* is a potential candidate species [1,15,16]. However, to extend the probiotic microorganisms “list” by adding bacteria originated from “unique” sources hold interests, as those species might harbor useful features of the microsystem where they are originated from and might form an attractive alternative to gut bacteria as they are evaluated from a safety perspective [17].

The characterization of the microbiota of wild tropical fruits draws our attention [14]. *Theobroma grandiflorum*, commonly known as white cacao, Amazonian cacao, or copoazú, is a perennial tree typical of the tropical humid forest, which has its origins in the Brazilian Amazon region and belongs to the *Malvaceae* family. Few reports have characterized the fruit chemical composition (i.e., high content of fatty acids in seeds) and the antioxidant and antimicrobial capacity, which could be of great interest in the food, pharmaceutical, and cosmetic industry [18,19]. In general, the pulp is used for the preparation of jams, juices, yogurt, nectars, and sweets. In Ecuador, this shrub is grown spontaneously near the Amazonian region (Orellana Provence, city of Napo) and the fruits are exploited by the indigenous population for the preparation of ceremonial drinks after a fermentation process or as part of typical dishes and consumed as fried chunks.

The microbiota of these fruits was not investigated. On the basis of large-scale screening and selection analysis, several *Lactobacillus* species were selected, and their biotechnological potential was assessed. Among them, the UTNGt2 strain showed high antimicrobial potential inhibiting both *E. coli* and *Salmonella enterica* at the exponential growth phase, demonstrating its bacteriolytic mode of action [16,20]. Peptide extracts from the UTNGt2 strain perforate the cytoplasmic membrane, which disturbs the cell integrity, leading to the release of intracellular molecules, and finally to cellular death. On the basis of molecular analysis, we showed that the peptide extract contains more than one peptide or protein-like substance which work together for the overall inhibitory activity. Moreover, coating the tomato fruits with peptide extracts-based suspension proves to be a promising approach to control spoilage growth, thus enhancing the safety of food products [16]. Additionally, the UTNGt2 strain induces spheroplast and filament formation along with cell vacuolation and DNA relaxation as a secondary killing event in *Salmonella enterica* [21].

Considering the high antimicrobial capacity of the UTNGt2 strain demonstrated in vitro and ex vitro, in the present research, we perform genomic sequencing and characterization to identify specific genetic features related to food safety and highlight specific features for possible application as probiotics and the antimicrobial peptides producer strain. A gene prediction with Prokka and functional annotation with EggNOG is performed to identify the potential involvement of the predicted genes in different biological pathways. By employing different available web server tools, the presence of CRISPR sequences, antibiotic-resistant genes, virulence, and pathogenicity factors is predicted. The detection of biosynthetic gene clusters of antimicrobial compounds is investigated using BAGEL 4 (http://bagel.molgenrug.nl/, accessed on 20 November 2020) web server, a versatile fast genome mining tool valid not only for modified-, and nonmodified bacteriocins, but also for non-bactericidal ribosomally produced and post-translationally modified peptides (RiPPs) [22]. The input FASTA contig is analyzed in parallel using antiSMASH (http://antismash.secondarymetabolites.org, accessed on 2 December 2020), a web server tool for the automatic genomic identification and analysis of biosynthetic gene clusters [23]. This genomic meaning tool allows for the prediction of PKs (polyketide), NRPs (non-ribosomally produced peptides), RiPP-like peptides, terpenes, covering a wide range of known or putative secondary metabolite compounds. Subsequently, the present study aims to get an insight into *L. plantarum* UTNGt2 genome to predict the functionality of the genes encoding for bacteriocins, for further use as potent antimicrobials producer and complement with the detection of several secondary metabolites for additional use as a probiotic strain.

## 2. Results and Discussion

### 2.1. General Genome Overview of UTNGt2 Strain

A total of 3,264,448 bp reads with N50 of 186,152 bases, generated from the genome library were assembled into 31 scaffolds using the SPAdes genome assembler. The assembly summary results are presented in Supplementary Table S1. Before assembly, a k-mer analysis was performed to estimate the genome size of the sample. The mean of the k-mer coverage was 218.8, and the genome size was estimated at 3,540,752 bases with 457,386 bases of genome repeats length and 0.029 overall rates of heterozygosity. The GC content was 44.53%. The genome of reference *L. plantarum* WCFS1 strain isolated from human saliva was estimated at 3,348,624 bases with 44.5% GC content. To validate the accuracy of the assembly, Illumina reads were mapped to the assembly results (Supplementary Table S2). To assess the completeness of the genome assembly, BUSCO analysis was performed on the basis of evolutionarily informed expectations of gene content from near-universal single-copy orthologs. The recovered matches were classified as complete if their lengths were within the expectation of the BUSCO profile match length. When found more than once, they were classified as duplicates. The matches that were only partially recovered were classified as fragmented, and BUSCO groups for which no matches passed the test of orthologs were classified as missing. The summary of BUSCO analysis results is shown in Supplementary Table S3. Higher complete BUSCOs indicate good assembly. After the complete genome was assembled, BLAST analysis was carried out to identify to which species each scaffold showed similarity. All 31 contigs analyzed matched the core genome of different strains of the genera *Lactobacillus* with 99% identity (Table 1). Because the BLAST analysis resides on registered information from the database, the assembly results are matched with a relative or evolutionarily distant species due to sequence differences or errors that may occur during the assembly process. Therefore, rather than using it as an absolute criterion for species determination, it was more appropriate to use the analysis to identify patterns. More recently, on the basis of conserved pairwise average amino acid identity, core genome phylogeny, physiological criteria, clade-specific signature genes, and ecology, the genus *Lactobacillus* was split into 25 genera including *Lactiplantibacillus*, *Fructiplantibacillus*, *Lactobacillus*, among others [24]. Thus, we allocated the study samples according to this new classification and the UTNGt2 was assigned as *Lactiplantibacillus plantarum.*

### 2.2. Phylogenetic Analysis

**Figure 1:**
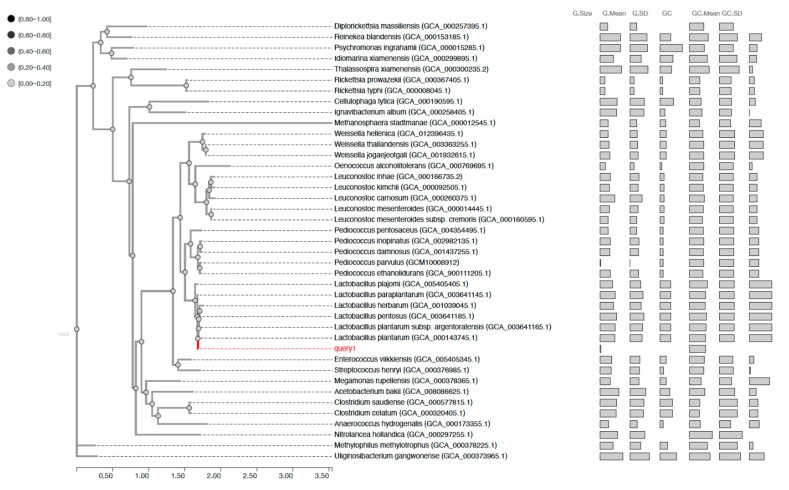
Phylogenetic tree based on the multiple alignment result of 56 selected marker genes with the gyType database. Query 1: UTNGt2 strain (red line).

The target sequence was aligned to the gcType 16S rDNA gene database, then several 16S rDNA sequences were selected to perform phylogenetic analysis on the basis of the alignment results.16S gene completeness was evaluated on the basis of the comparison between the submitted sequence and its most similar sequence in the gcType 16S rDNA gene database. A phylogenetic tree established with 16S rDNA analysis is presented in Supplementary Figure S1. As most of the used phylogenetic methods were developed to infer the phylogeny of a gene, and many genes have undergone horizontal transfer events, to elucidate precise phylogenetic relationships among genomes, a generally accepted process to infer whole-genome phylogeny is to use multiple genes that are thought to be orthologous (or unlikely underwent lateral transfer events) [25]. The genome of UTNGt2 was compared with selected 40 genomes from Firmicutes phylum. A list of reference genomes, species, and lineage is presented in Supplementary Table S4. To perform marker gene-based phylogenetic analysis, 56 marker genes were extracted from the submitted and selected genomes. The alignment results of the 56 marker genes are shown in Figure 1. This analysis placed the UTNGt2 strain in the same clade with *Lactobacillus plantarum* (GCA_000143745.1), *L. plantarum* subps. *argentoratensis* (GCA_0013641165.1), and *L. pentosus* (GCA_003641185.1).

### 2.3. Gene Prediction and Genome Annotation

**Figure 2:**
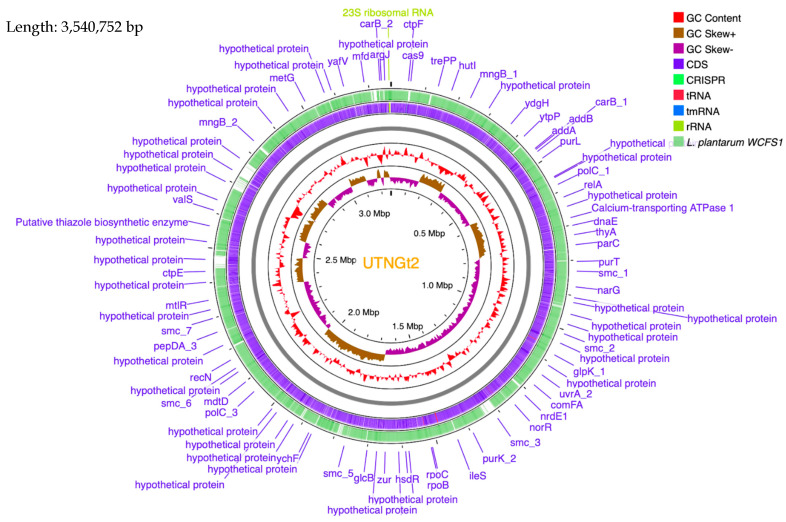
Circular map view of the *L. plantarum* UTNGt2 genome generated using CGView Server (http://cgview.ca, accessed on 16 February 2021). The contents are arranged in feature rings (starting with outermost ring): outermost first ring represents the map of reference taxa *Lactobacillus plantarum* WCFS1; second ring shows the UTNGt2 CDS (coding sequences) with Prokka annotation (both strands combined); tRNA, rRNA, tmRNA are indicated; third ring displays the G + C content; fourth ring G/C skew information in the (+) strand (brown color) and (−) strand (violet color); CRISPR are marked.

**Figure 3:**
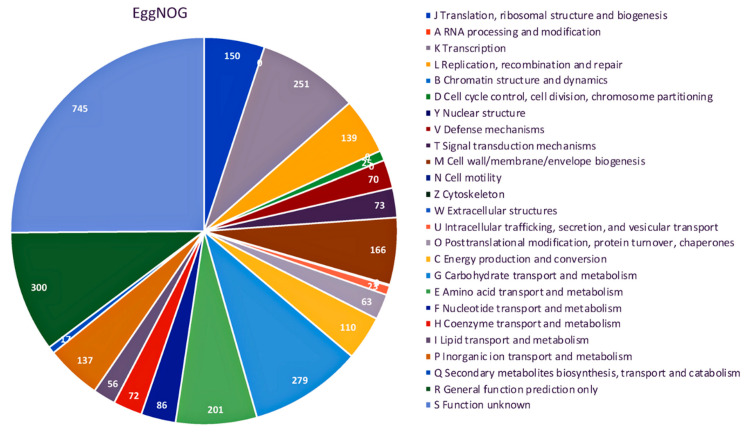
EggNOG category distribution of functional annotation result. Number of genes per category is indicated.

**Figure 4:**
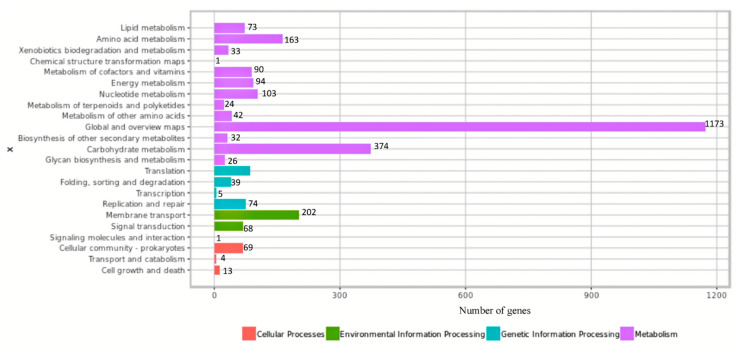
Mapping of the KEGG proteins according to their function. The number of genes in each category is shown.

The genome contains 3115 genes, 3052 protein-coding genes (CDS), 57 tRNAs, 5 rRNA, and 1 copy of tmRNA. A physical genome map of UTNGt2 in comparison to the reference strain *L. plantarum* WFCS1 is shown in Figure 2. Prokka was used to predict the location, while BLAST was used to know about the function and identification of assembled sequences against nucleotide and protein sequence database. Based on the EggNOG annotation results, 9.4% of proteins are involved in carbohydrate transport and metabolism, 8.46% in transcription, 2.36% responsible for defense mechanisms, 0.5% responsible for the biosynthesis of secondary metabolites, transport, and catabolism, while 25.11% has an unknown function (Supplementary Table S5). The genes encoding proteins and their predicted functions are displayed in Figure 3. Predicted genes with Prokka in the previous step were aligned with several databases to obtain their corresponding annotations with the aligners. The summary of the annotation results is shown in Table 2. Genes were annotated with the KEGG protein ID first, then mapped to the KO entry, and further mapped to the KEGG pathway. The number of genes in each category is shown in Figure 4. The production of vitamins during the fermentation of raw material by lactobacilli is greatly exploited by food biotechnology. Based on Prokka and EggNOG annotation, UTNGt2 harbored folate (vitamin B9) and riboflavin encoding genes and is deficient in the genes encoding for niacin (B3), pantothenate (B5), and pyridoxine (B6). However, the riboflavin cluster contains 7 genes (*ribBA*, *ribD*, *ribE*, *ribH*, *yitU*, and *ribF* (2 copies)), and the folate cluster contains 6 genes (*folB*, *folC*, *folD*, *folE*, *folK*, *folP*). Early investigation showed the presence of nine folate genes in the reference strain *L. plantarum* WCFS1 genome. The riboflavin cluster genes were found early in several strains such *L. plantarum* ATCC8014, KC28, JDM1, and so forth. [1]. Recently, fortifying different natural food matrices with UTNGt2 cells increases Vitamin C content, total polyphenols, and antioxidant capacity (GNT, unpublished results), suggesting that that lactic cells could enhance the bioavailability and antioxidant capacity of these natural matrices, a benefit given by a probiotic strain.

### 2.4. Prediction of Antibiotic Resistance Genes, Virulence Factors, Pathogenicity, and Prophage

On the basis of the CARD protein ID, 39 predicted genes were grouped by Drug Class, Resistance Mechanism, and AMR gene family. The number of genes in each category is shown in Supplementary Figure S2A–C). The UTNGt2 strain harbored several putative resistance genes associated with resistance to lincosamide (6), penam (4), tetracycline (4), fosfomycin (5), fluoroquinolone (8), a macrolide (3), rifamycin (3), and peptide (11). On the basis of RGI tool criteria, any antimicrobial-resistant genes were detected. Recent research on five *L. plantarum* strains isolated from fermented chicha beverage indicated the presence of *aadA* gene encode for aminoglycoside modifying enzymes which mediate specifically the resistance against spectinomycin and streptomycin [1]. In the present study, by using CARD analysis, RGI, and Prokka no equivalent intrinsic aminoglycoside resistance genes were predicted. Complementary antibiotic susceptibility (antibiotic disk assay and minimum inhibitory concentration value determination) according to the FEEDAP document [26] indicate that UTNGt2 is susceptible to ampicillin, amoxicillin, gentamycin, erythromycin, penicillin, cefotaxime, and tetracycline (Supplementary Table S6) as any MIC values were higher than the breakpoints. The presence of Tet-R gene in *L. plantarum* B43 isolated from the human oral cavity was previously investigated [27]. The most common Tet-R genes (*tet*M, *tet*W, *tet*K, *tet*L, *tet*S, and *tet*O) code a RPP (ribosomal protection proteins) or MFS (major facilitating superfamily) efflux protein. Through whole-genome analysis with Prokka and EggNOG annotation, two tetracycline-resistant genes, *tetO* (COG0408) and *tetA* (tetracycline-resistant protein class B), have been detected in the UTNGt2 strain. BLASTN analysis indicated 99.75% identity with *L. plantarum* (WP_024002387.1). Early studies on *L. plantarum* CCUG 43,738 indicated that the strain displayed atypical phenotypic resistance to tetracycline (MIC, 512 μg/mL) due to the presence of *tet*(S) gene located on a plasmid [28]. In other studies, the presence of *tetW* gene in *Bifidobacterium animalis* subsp. *lactis* was associated with tetracycline resistance [29]. No acquired resistance genes were found in the UTNGt2 genome, neither mobile elements were detected with PlasmidFinder indicating its stability.

Several genes (a total of 103 genes predicted, from which 11 were not annotated) were predicted as putative virulence factors by VFDB (data not shown). Any of the known virulence genes *gelE* (gelatinase), *hyl* (hyaluronidase), *asa1* (aggregation substance), *esp* (enterococcal surface protein), *cylA* (cytolisin), *efaA* (endocarditis antigen), and *ace* (adhesion of collagen), were detected in the UTNGt2 genome. The absence of these genes was confirmed by EggNOG annotation results. Two genes (*sugC*) coding for carbohydrate ABC transporter ATP-binding protein (CUT1 family) with 46.8% and 52.3% identity, a gene coding for putative enolase (*eno*) with 70% identity, and a putative gene coding for hemolysin III (*hlyIII*) with 42.4% identity, were predicted in the category of virulence factors, but their expression needs to be further investigated. Early genome comparison of several *L. plantarum* strains indicated the presence of several putative genes annotated as virulence factors (such as the putative formate acetyltransferase 3-ybiW gene or *mdxE*-maltodextrin (ABC-transporter protein gene), but they seem to be only spurious partial hits, which may exert other cellular functions [30]. In this study, the *mdxE* gene, but not 3-ybiW, was annotated with EggNOG in the UTNGt2 genome in the category “G” of carbohydrate transport and metabolism.

Analysis using contigs as input files exhibited that the strain does not contain any pathogenicity factors. The probability of being a pathogen was low (<0.081 out of 1) and the strain was predicted as a nonhuman pathogen. These results were consistent with the qualified presumption of the safety status and agreed with other studies indicating that five *L. plantarum* strains isolated from a fermented chicha, an indigenous drink from Argentina were safe [1].

PHASTER analysis revealed the presence of four prophages, from which only one intact region was predicted in the contig 6, while the other three were incomplete and detected within contigs 2, 3, and 5 (Supplementary Table S7). The highest intact phage region has 39.9 Kb with the most common: PHAGE_Lactob_Sha1_NC_019489(27). A similar intact phage was detected in *L pentosus* MP-10 genome [31] and several *L plantarum* strains [30]. The incidence of prophage DNA (>40) within lactobacilli genomes were reported and their presence highlights the genetic diversity and fitness of the *Lactobacillus* genome [31], as well as may confer a selective advantage to the cell, promoting its survivability and its resistance to other infecting phages [32]. On the basis of these results, we conclude that the UTNGt2 strain might be considered a safe strain.

### 2.5. CRISPR/Cas System Prediction

The genome of UTNGt2 strain harbor one CRISPR arrays within contig 1 (begin at 50,489) with 36 bp repeat length and 11 repeats matching a consensus sequence with evidence level 4 according to CRISPRFinder analysis (Table 3). Three additional putative CRISPR arrays were detected within contigs 8 and 9 (evidence level 1). One mandatory CRISPR-associated protein of Type IIA system (Csn2_0_II_A), and three CRISPR-associated endoribonucleases, Cas2_0_I-II-III, Cas1_0_II, and Cas9_0_II of Type IIC system were predicted within contig 1 (Table 4). These genes were annotated with Prokka and EggNOG with the category “L” of Replication, recombination, and repair. Compared with other well-characterized main types such as I and III, the Type II systems are rare in nature and possess three subtypes, II-A, II-B, and II-C, and two variants, II-C1 and II-C2 [33]. The Cas9 proteins represent the signature protein of these systems. Early research indicates that Cas9 has some involvement in new spacer acquisition [34] and may prime potential protospacer sequences for Cas1/Cas2 recruitment [35]. Nonetheless, CRISPR/Cas system may prevent the *L. plantarum* strains to acquire antimicrobial-resistant genes or pathogenic genes through horizontal transfer genes [1]. By employing CRISPRCasFinder web service, in the reference strain *L. plantarum* WCFS1, no CRISPR array was detected. Nonetheless, along with the prediction of probiotic capabilities, there is an increasing interest in the manipulation of the genome of probiotic strains via the CRISPR/Cas system for the production of the next generation of robust probiotics with novel functionalities and the capacity to deliver new biomolecules.

### 2.6. Prediction of Bacteriocins and Bioactive Products

**Figure 5:**
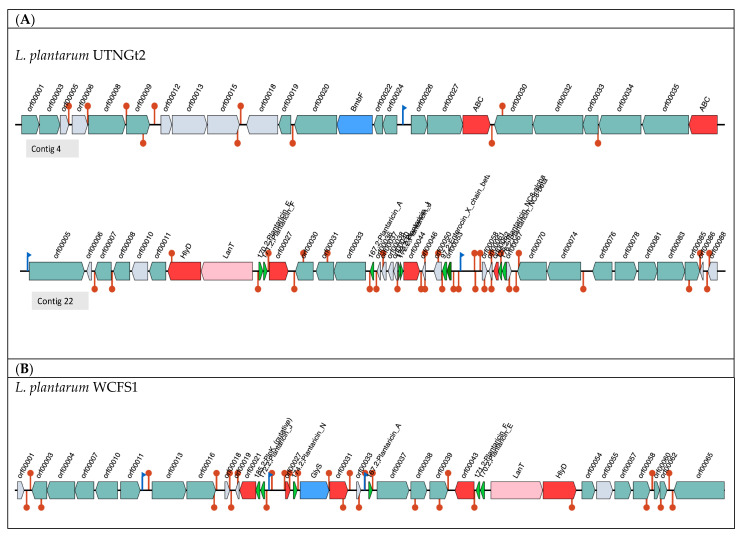
Organization of bacteriocin cluster genes. Area of interest (AOI) of *L. plantarum* UTNGt2 of contig 4 and contig 22 (**A**) and *L. plantarum* WCFS1 (**B**). Genes with function determined from left to right (gene name, function, and locus tag): contig 4: Probable ABC transporter permease protein and Probable ABC transporter ATP-binding protein spyM18_0273; GTP 3′,8-cyclase OS = *Lactobacillus plantarum* (strain ATCC BAA-793/NCIMB 8826/WCFS1); contig 22: orf00005, DNA helicase IV OS from *Bacillus subtilis* (strain 168); orf0007, orf0008 and orf00011, PlnS (CAAX protease self-immunity); HlyD, accessory factor for ABC-transporter PlnH; LanT, bacteriocin ABC-transporter, ATP-binding and permease protein PlnG; 170.2 Plantaricin_F, plantaricin F; orf00027, P71468_LACPL PlnI (Immunity protein PlnI, membrane-bound protease CAAX family); orf00030, response regulator PlnD; orf00031, response regulator PlnC; orf00033, bacteriocin production related histidine kinase; 167.2 plantaricin A, orf00036-00040, function not determined; orf00044, putative bacteriocin Immunity protein; orf00046, orf00050, not determined function; 97.2 Enterocin_X_chain + beta; orf00053, ComC; Lactococcin; Bacteriocin_IIc; 176.2;Plantaricin_NC8-beta; 171.2; Plantaricin_F; 175.2;Plantaricin_NC8-alpha; 172.2;Plantaricin_J. GlyS: Glycotransferase family 2 protein; 185.2, PlnK putative; 174.2, Plantaricin N (bacteriocin 2c); red blocks: immunity and transport; green arrow: core peptide; pink block: transport and leader cleavage; blue block: peptide modifications; grey blocks: no function determined.

Antimicrobial activity against foodborne pathogens was previously described [20]. Cell-free supernatant from UTNGt2 showed high inhibitory potential against *Salmonella* spp., *Shigella* spp., *E. coli* spp., and *Enterobacter* spp., and the inhibitory activity was proved to be of proteinaceous demonstrated by the presence of different size peptides produced in the extract [20]. Additionally, the potential of this strain to inhibit pathogens and fungi was demonstrated in tomato fruits. In this study, by employing BAGEL 4 webserver, we detected that UTNGt2 harbors two bacteriocin clusters, named as Area of interest (AOI) within contig 4.12 (start at 63,041, end 83,041) of the sactipeptide class (ribosomally synthesized peptides), and contig 22.27 (start at 11,549, end 41,159) of plantaricin E class (Figure 5). The AOIs located in contig 4 consist of two ABC transporter ATP binding proteins, and multiple ORFs (Open Reading Frames)**,** while the AOI of contig 22 resides of plantaricin E/F, plantaricin J (2 protein), plantaricin A, as core proteins. Further, two genes, HlyD and LanT coding for Accessory factor for ABC-transporter PlnH and Bacteriocin ABC-transporter, ATP-binding, and permease protein PlnG were detected downstream of plantaricin E/F (class IIb two-peptide bacteriocin)**.** Three additional ORF regions, orf000033, orf00044, and orf00062 were predicted as Bacteriocin production-related histidine kinase and putative bacteriocin immunity proteins, respectively. Plantaricin A belongs to the alpha/beta Enterocin family, which is a class II bacteriocin with double-glycine leader peptide (Evalue = 3 × 10^−29^, match = 100.00%). Enterocin X_chain_beta protein sequence from UTNGt2, is a lactococcin-like protein (class IIb), having 53% identity with enterocin X from *Enterococcus faecium* KU-B5. BLASTN analysis indicated that this peptide was found in *Lactiplantibacillus plantarum* (WP_027821506.1) with 100% amino acid sequence identity. These peptide does not show any conserved domain. Enterocin X is a thermostable non-lanthionine-containing two-peptide bacteriocin whose full antibacterial activity requires the interaction of two complementary peptides [36]. Early genome analysis of *L. plantarum* strain CECT8963 isolated from chicha fermented beverage showed different organization of bacteriocin-like genes, neither enterocin X, plantaricin NC8-alpha and NC8-beta, plantaricin A and J were detected [1].

The reference strain *L. plantarum* WCFS1 strain harbors a single bacteriocin cluster as detected by BAGEL 4 analysis and the orientation of the genes was opposite to the same genes found in UTNGt2. Database hit to plantaricin J, putative plnK, plantaricin A, plantaricin N, and plantaricin E/F along with several ORFs coding for ABC transporters. However, the bacteriocin cluster of UTNGt2 strain is more complex organized harboring several genes coding for different bacteriocins, which might explain its broad inhibitory spectrum [20]. Molecular analysis indicated four products of approximately 22, 32, 35, and 55 kDa in the peptide extract from UTNGt2 [16]. On the basis of EggNOG annotation, two additional genes, *lcnD* and *lacD* coding for Lactococcin A secretion protein LcnD and Lactococcin-G-processing and transport ATP-binding protein LagD were detected. BLAST against data core proteins indicated that the UTNGt2 genome sequence hit with rSAM-modified_RiPP_077 (Bit Score = 33.113), rSAM-modified_RiPP_075 (Bit Score = 31.187), bovicin_225_variant (Bit score = 32.7278) from *Streptococcus bovis* and Blp (Bit score = 30.0314) from *Streptococcus pneumoniae* TIGR4. These findings were in agreement with our previous results indicating the high inhibitory potential of this strain as more than one peptide act as an antimicrobial unit [21]. By treating *Salmonella* cells with UTNGt2 peptide extract at the final concentration of 1 X MIC, spheroplast formation was observed, the cells showed changed shape, the inner and outer membranes were intact, but they lost peptidoglycans layer, while by increasing the concentration of peptides (2 X MIC) along with the spheroplasts, some “ghost cells” were noted, indicating that target bacteria were devoid or near-devoid cytoplasm [21]. An early study on conventional antibiotics, such as penicillin G, and beta-lactam, indicated that these convert bacteria into spheroplasts [37,38]. Additionally, the production of ghost cells might be exploited further for the production of the next generation of vaccines.

**Figure 6:**
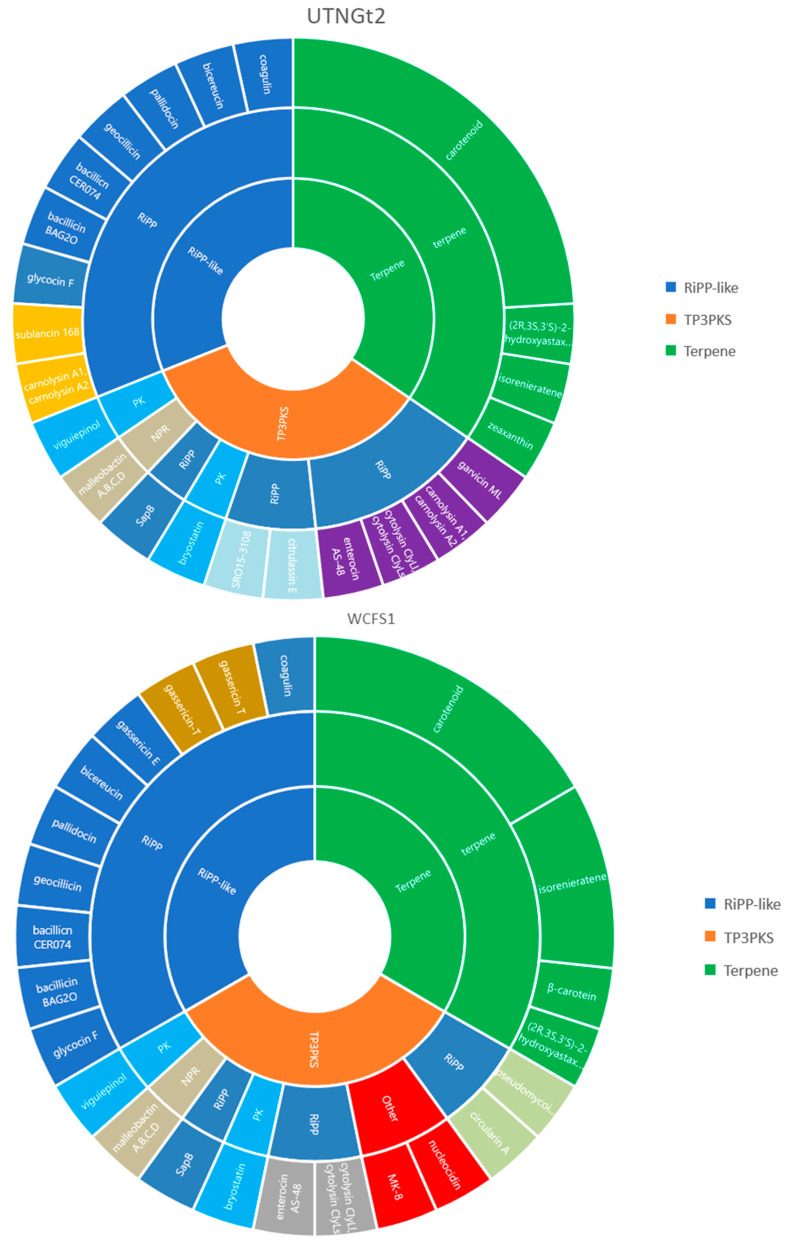
A comparison between the RIPP gene clusters detected in the UTNGt2 genome and the reference *L. plantarum* WCFS1 strain. Different color marked the difference between the substances produced by each strain. Legend: RiPP-like: other unspecified ribosomally synthesized and post-transcriptionally modified peptide product (RiPP) cluster; RiPP: Ribosomal synthesized and post-translationally modified peptides; T3PKS: Type III polyketide synthase; Terpene: terpene compounds. PK: polyketide; NRP: non-ribosomally produced peptides. Other: cluster containing a secondary metabolite-related protein that does not fit into any other category.

Microorganisms can produce a diverse range of metabolites known as natural products encoded by different biosynthetic gene clusters. Ribosomal synthesized and post-translationally modified peptides (RiPPs) are defined by a genetically encoded precursor peptide that is tailored by the associated biosynthetic enzymes to form the mature product [39]. These metabolites had various biological functions [40]. Two major metabolites biosynthetic clusters of bacteria enclose polyketide synthases (PKS) and non-ribosomal peptide synthases (NRPS), which are well-known to synthesize a diverse range of products with beneficial applications in medicine and research, such as antibiotics, antifungals, and immunosuppressants [41]. In this study, using AntiSMASH webserver, three metabolites’ regions were detected as follows: region 6.1 (location:1–26,879 nt): T3PKS (Type III polyketide synthase) within contig 6, region 16.1 (location: 26,176–47,057 nt): Terpene, contig 16 and region 22.1 (location: 14,131–26,281 nt): RiPP-like proteins within contig 22. When analyzing the most similar gene clusters by comparison with MiBIG database, several cluster regions were detected (Supplementary Table S8). A comparison between the RIPP gene clusters detected in the UTNGt2 genome and the reference *L. plantarum* WCFS1 strain is shown in Figure 6. By employing similar web tools, early studies indicated that the genome of *L. plantarum* strain LL441 does not harbor any putative bacteriocin gene cluster or genes involved in the synthesis of secondary metabolites with an antimicrobial capacity [15]. Thus, the results indicated that the UTNGt2 strain can be used as a bacteriocin producer strain and as a source of bioactive natural products.

## 3. Materials and Methods

### 3.1. Strain and Culture Conditions

*Lactiplantibacillus* (formally *Lactobacillus*) *plantarum* strain UTNGt2 was grown in MRS medium (Merck, Darmstadt, Germany) at 37 °C under static aerobic conditions. The strain was previously identified based on 16S rDNA sequencing and was registered in 2018 (November) with GenBank accession no. KY041688.1.

### 3.2. NGS de Novo Assembly of UTNGt2

De novo assembly was performed to generate sequences of UTNGt2 sample using Illumina HiSeq X Ten platform as previously described (custom service, Macrogen Inc.; Seoul, Korea) [42]. In brief, the sequencing library was prepared by random fragmentation of the DNA or cDNA sample, followed by 5′ and 3′ adapter ligation according to the protocol provided by the manufacturer (Macrogen Inc.; Seoul, Korea). Sequencing data were converted into raw data for analysis. The overall quality of reads generated by FastQC (v0.11.5, http://www.bioinformatics.babraham.ac.uk/projects/fastqc, accessed on 16 October 2020), total bases, total reads, GC content, and basic statistics were calculated. To reduce biases in the analysis, adapter trimming and quality filtering were performed. Trimmomatic v0.36 (http://www.usadellab.org/cms/?page=trimmomatic, accessed on 16 October 2020) was used to remove adapter sequences. The quality of filtered reads, total bases, total reads, GC content, and basic statistics were calculated. De novo assembly was performed by various k-mer using SPAdes 3.15.1 (http://cab.spbu.ru/software/spades/, accessed on 16 October 2020). K-mer analysis was performed to provide information about coverage, heterozygosity, and estimated genome size (Jellyfish v2.2.10, http://www.genome.umd.edu/jellyfish.html, accessed on 16 October 2020). Using filtered reads, de novo assembly was performed using a De Bruijn graph assembler (http://qb.cshl.edu/genomescope/, accessed on 16 October 2020). The reads were split into multiple K-mers (Oligonucleotides with length “K”, K equal integer), which were further aligned with each other, and the sequences were extended one base at a time based on the overlap information of K-mers. The best k-mer was selected on the basis of various statistics from assembly results (number of contigs, total base of contigs, N50, etc.) and the best-assembled sequence set was determined. The assembled genome was validated using mapping strategy and BUSCO analysis (https://busco.ezlab.org/, BUSCO version 3.0) [43]. The filtered reads were aligned to the assembled genome, and their insert size was estimated for validation. To assess the completeness of the genome assembly, BUSCO analysis was performed based on evolutionarily informed expectations of gene content from near-universal single-copy orthologs. By default, a bacteria database was used for analysis. After the complete genome or draft genome was assembled, BLAST analysis was carried out to identify to which species each scaffold showed similarity.

### 3.3. Evolutionary Relationship

The total contig sequences in FASTA format were submitted to the gcType whole-genome database through the Global Catalog of Microorganisms genome annotation project pipeline initiative (http://gctype.wdcm.org) [44] for species classification. Based on the web tool analysis strategy, from the submitted genome sequence the 16S rDNA sequence(s) is extracted and aligned to the gcType 16S rDNA gene database using BLAST. BLAST results are sorted in descending order based on the identity (i.e., the top sequence is the one with the highest sequence similarity) and are used to estimate the 16S rDNA gene completeness. Extracted and selected 16S rDNA sequences are further aligned using MAFFT [45]. By default, RAxML (Randomized Axelerated Maximum Likelihood) was employed for phylogenetic analysis of large datasets under maximum likelihood [46]. Its major strength is a fast maximum likelihood tree search algorithm that returns trees with good likelihood scores. Moreover, the submitted genome sequence is aligned to the gcType whole-genome database using Mash [47]. Various genome similarity metrics, Average Nucleotide Identity between the genomes (ANIb), FastANI (fast alignment-free computation of whole-genome Average Nucleotide Identity), orthoANIb, and orthoANIu were calculated between the submitted genome and genome sequences from the database [25,48,49]. By default, for phylogenetic analysis, a multi-marker association method for genome-wide association studies without the need for a population structure correction was assessed [50].

### 3.4. Gene Prediction and Genome Annotation

After the whole genome or draft genome was assembled, the location of protein-coding sequences, tRNA genes, rRNA genes, and tmRNA genes was identified. Then, their functions were annotated. The following tools were used to predict each functional element: Prodigal for CDS prediction [51], RNAmmer for rRNA prediction (http://www.cbs.dtu.dk/services/RNAmmer/, accessed on 16 October 2020), Aragorn for tRNA/tmRNA prediction (http://www.ansikte.se/ARAGORN/, accessed on 16 October 2020), Signal IP for signal leader peptide prediction (http://www.cbs.dtu.dk/services/SignalP/, accessed on 16 October 2020) and Infernal for non-coding RNA prediction. After gene prediction was completed with the Prodigal prediction of protein-coding sequences, ORFs, and subsequent gene annotation, was performed using the Microbial Genome Annotation Pipeline online server (Prokka v1.14.5), by which coding sequences and rRNAs and tRNAs were predicted [52]. Prokka conducts analysis based on ISfinder, NCBI Background Reference Gene DB, UniProtKB DB, and HMM DB. Genome map of *L. plantarum* UTNGt2 was predicted using CGView [53]. For comparison, reference strain *Lactobacillus plantarum* WCFS1 and the complete sequence genome available in the NCBI database were used (NC_004567.2).

### 3.5. Functional Annotation

For functional annotation, InterProScan v5.0 (https://www.ebi.ac.uk/interpro/search/sequence/, accessed on 16 October 2020) and EggNOG DB (Evolutionary genealogy of genes: Non-supervised Orthologous Groups), provided by EMBL (http://eggnog5.embl.de/#/app/home, accessed on 16 October 2020) were used [54,55]. EggNOG DB is similar to COG DB, which contains genes classified on the basis of their function and annotations. In this analysis, psi-BLAST was used to match the predicted protein sequences with EggNOG DB. Moreover, through the Global Catalog of Microorganisms genome annotation project pipeline (http://gctype.wdcm.org, accessed on 25 November 2020) [44], the predicted genes were annotated automatically by several databases, SwissProt (https://www.uniprot.org/statistics/Swiss-Prot, accessed on 25 November 2020), MetaCyc (a database that contains pathways responsible for both primary and secondary metabolism, as well as associated metabolites, reactions, enzymes, and genes; https://metacyc.org, accessed on 25 November 2020); KEGG (Kyoto Encyclopedia of Genes and Genomes); CAZy: Carbohydrate-active enzyme (http://www.cazy.org/, accessed on 25 November 2020); VFDB (a virulence factor database, http://www.mgc.ac.cn/VFs/main.htm, accessed on 25 November 2020); PHI: The Pathogen-Host Interaction database is a biological database that contains curated information on genes experimentally proven to affect the outcome of pathogen-host interactions (http://www.phi-base.org/searchFacet.htm?queryTerm, accessed on 25 November 2020).

### 3.6. Prediction of Genes Involved in Food Safety

The genome was assessed for safety using several tools recommended in the EFSA Guidance [11]. CARD (Comprehensive Antibiotic Resistance Database) (http://arpcard.mcmaster.ca/, accessed on 13 January 2021) [56] for identifying antibiotic resistance and virulence factors using RGI tool (Resistance Gene Identifier) (under Perfect hit, Rigorous hit alone, and Perfect, Strict, and Loose hit criteria) was used by importing the contigs with “perfect and strict hits only” and “high-quality coverage” [57]. The ResFinder 4.1 server (https://cge.cbs.dtu.dk/services/ResFinder/, accessed on 13 January 2021) was employed to identify the acquired antimicrobial resistance genes with a selected % ID threshold of 90.00% and the Selected minimum length of 60% and/or chromosomal mutations [58]. PlasmidFinder 2.0 was used to search for mobile elements (https://cge.cbs.dtu.dk/services/PlasmidFinder-2.0/, accessed on 13 January 2021); VFDB (virulence factor database, http://www.mgc.ac.cn/VFs/main.htm, accessed on 13 January 2021) was employed to predict putative virulence factors [59]. CRISPRFinder (https://crisprcas.i2bc.paris-saclay.fr/CrisprCasFinder/Index, accessed on 13 January 2021) was used to detect the CRISPR and Cas genes by importing the contig FASTA sequences and selecting “Perform cas detection” and leaving the rest of the parameters by default [60]. PHASTER (PHAge Search Tool Enhanced Release) (http://phaster.ca, accessed on 15 January 2021) [57] were used to detect and annotate prophage sequences within bacterial genomes. PathogenFinder web server (http://cge.cbs.dtu.dk/services/PathogenFinder/, accessed on 15 January 2021) was used for the prediction of bacterial pathogenicity using as input the contig file in FASTA format [61].

### 3.7. Prediction of Putative Gene Cluster Coding Bacteriocins and Other Bioactive Compounds

To predict genes coding for bacteriocins and ribosomal synthesized and post-translationally modified peptides (RiPPs), the BAGEL4 webserver 6 was used by importing the contig FASTA sequences [62]. Also, BLAST analysis against the core peptide database was performed. To identify the gene clusters encoding secondary metabolites of all known broad chemical classes, AntiSMASH version 6.01 alfa 1 (Antibiotics and Secondary Metabolite Analysis Shell) [23] webserver (https://docs.antismash.secondarymetabolites.org, accessed on 2 December 2020) was used. The contigs FASTA file of the UTNGt2 was used as the input file, selected by default antiSMASH features: “KnownClusterBlast analysis”: the identified clusters are searched against the MIBiG repository (a hand-curated data collection of biosynthetic gene clusters, which have been experimentally characterized) and “Subcluster Blast analysis”: the identified clusters are searched against a database containing operons involved in the biosynthesis of common secondary metabolite building blocks (e.g., the biosynthesis of non-proteinogenic amino acids) as well as detection of the active sites (active sites of several highly conserved biosynthetic enzymes are detected and variations of the active sites are reported). An overview of the detected regions in the contigs is displayed as recommended [63].

## 4. Conclusions

Overall, this study reports the whole-genome sequence of *Lactiplantibacillus plantarum* UTNGt2 strain, being to the best of our knowledge the first genome draft of a lactic bacteria isolated from wild cacao fruit. This unique genome was composed of several genes encoding for proteins with unknown or hypothetical function. On the basis of genomic analysis, various protein families were categorized according to their functions, and cluster genes of secondary metabolites were detected. However, two bacteriocin cluster genes and several RiPP-like peptides, polyketides, and terpenes were detected, thus confirming its promising antimicrobial capacity or a novel therapeutic agent. The genetic variability of *L. plantarum* UTNGt2 strain was defined by the diversity of hypervariable CRISPR/Cas systems. The production of riboflavin and folate, the absence of acquired antibiotics resistance genes, virulence, and pathogenic factors suggesting that UTNGt2 is a safe strain, thus can be further tested for its probiotic potential and production of bioactive compounds (enzymes, vitamins, amino acids, etc.) for the food industry. Our study illustrated the genome of a strain with valuable traits to be additionally exploited as a natural food preservative or protective cover that might reduce the contamination by pathogenic microorganisms invading the food during manipulation or storage. The dual role, probiotic and antimicrobial, will allow to design novel antimicrobial formulations, peptide- or cell-based, for a novel starter or preservative culture in dairy or no dairy foods, giving, as a result, a fortified product with acceptable organoleptic profile, with improved characteristics such a high inhibitory ability, prolonged shelf-life, or additional bioactive health-promoting features including, enhanced antioxidant capacity and improved vitamins and polyphenols content, inherent to their ecological origin, which could, thus, be explored in various biotechnological applications.

## Figures and Tables

**Table 1:** Description of the best hit results matches in the NCBI NT database.

Accession	Description	Query #	Query Length	I_Pct. (%)
CP013750.1	CP013750.1 *Lactobacillus plantarum* strain KP plasmid unnamed1, complete sequence	1	87,144	99
CP009236.1	CP009236.1 *L. plantarum* strain 5–2, complete genome	4	436,043	99
CP025991.1	CP025991.1 *Lactiplantibacillus plantarum* subsp. *plantarum* strain LB1-2 chromosome, complete genome	1	63,686	99
CP012343.1	CP012343.1 *L. plantarum* strain ZS2058, complete genome	1	48,000	99
CP034694.1	CP034694.1 *Lactiplantibacillus plantarum* strain FBL-3a chromosome, complete genome	17	1,859,159	99
CP031771.1	CP031771.1 *Lactiplantibacillus plantarum* subsp. *plantarum* strain E1 chromosome	1	1169	99
CP021932.1	CP021932.1 *Lactiplantibacillus plantarum* strain TMW 1.1478 chromosome, complete genome	1	249,265	99
CP035143.1	CP035143.1 *Lactiplantibacillus plantarum* strain SRCM103357 chromosome, complete genome	1	1748	100
CP046262.1	CP046262.1 *Lactiplantibacillus plantarum* strain KCCP11226 chromosome, complete genome	1	4509	100
CP031702.1	CP031702.1 *Lactiplantibacillus plantarum* strain IDCC3501 chromosome, complete genome	1	10,237	100
CP032464.1	CP032464.1 *Lactiplantibacillus plantarum* strain ATG-K6 chromosome, complete genome	1	260,051	99
CP023728.1	CP023728.1 *Lactiplantibacillus plantarum* strain 10CH chromosome, complete genome	1	243,437	99

Query #: number of the sequenced match; Query Length: query sequence length; I_Pct. (%): percentage of identical matches.

**Table 2:** Gene annotation summary performed by aligning gene sequences of the UTNGt2 genome to the sequences from several databases.

Sample	Number of Genes	Number of CDS	CARD	MetaCyc	PHI	CAZy	VFDB	SwissProt	KEGG	COG
Gt2	3155	3052	39 (1.27%)	413 (13.49%)	113 (3.69%)	107 (3.50%)	103 (3.36%)	1129 (36.88%)	2989 (97.65%)	2334 (76.25%)

Legend: CARD: Comprehensive Antibiotic Resistance Database; MetaCyc: database that contains pathways involved in both primary and secondary metabolism; PHI: The Pathogen-Host Interaction database is a biological database that contains curated information on genes experimentally proven to affect the outcome of pathogen-host interactions (http://www.phi-base.org/searchFacet.htm?queryTerm, accessed on 25 November 2020); CAZy: Carbohydrate-active enzyme (http://www.cazy.org/), accessed on 25 November 2020; VFDB: virulence factor database, (http://www.mgc.ac.cn/VFs/main.htm, accessed on 25 November 2020); SwissProt: https://www.uniprot.org/statistics/Swiss-Prot (accessed on 25 November 2020); KEGG: Kyoto Encyclopedia of Genes and Genomes; COG: Clusters of Orthologous Groups of proteins. Number of genes and % is shown.

**Table 3:** CRISPR array system detection with CRISPRFinder within the UTNGt2 genome.

CRISPR_ID	Start	End	Length (bp)	Potential Orientation	Consensus_Repeat	Number of CRISPRs_with_Same_Repeat (CRISPRdb)	Repeat_Length	Spacers Number	Number Repeats Matching Consensus	Conservation_Repeats (% Identity)	Evidence_Level
contig1_1	50,489	51,250	761	Reverse	GTTCTAAACCTGTTTGATATGACTACTATTCAAGAC	3	36	11	12	100.00	4
contig8_1	73,175	73,277	102	Reverse	ATGGCGAAGAAGAAGAACCAGAATACGACAAACC	0	34	1	1	97.06	1
contig8_2	150,018	150,117	99	Unknown	TGCACTGGTAACTGAGCTGGCGCTG	0	25	1	1	96.00	1
contig9_1	97,887	97,972	85	Reverse	AATAAGATACTTTAAGTTTCTTA	0	23	1	1	95.65	1

**Table 4:** Cas type detection with the CRISPRFinder webservice.

Sequence ID	Cas-Type/Subtype	Gene Status	System	Type	Begin	End	Strand
contig1_50	Csn2_0_IIA	Mandatory	CAS-TypeIIA	CDS	51,275	51,952	-
contig1_51	Cas2_0_I-II-III	Accessory	CAS	CDS	51,949	52,254	-
contig1_52	Cas1_0_II	Accessory	CAS-TypeIIC	CDS	52,232	53,137	-
contig1_53	Cas9_0_II	Accessory	CAS-TypeIIC	CDS	53,331	57,407	-

## Data Availability

Genome assembly data were deposited in the NCBI Database: BioProject PRIJNA705232 y BioSample SAMN18053630 on 26 February 2021.

## Supplementary Materials

The following are available online at https://www.mdpi.com/article/10.3390/antibiotics10040383/s1, Supplementary Table S1: Assembly summary results; Supplementary Table S2: Mapping overall results; Supplementary Table S3: BUSCO analysis results; Supplementary Table S4: List of reference ID genomes, species, and lineage used for phylogenetic analysis retrieved from the gyType database; Supplementary Table S5: EggNOG category distribution result; Supplementary Table S6: Antibiotic susceptibility of the UTNGt2 strain; Supplementary Table S7: Summary of Prophage regions identified on the UTNGt2 genome using the PHASTER webserver; Supplementary Table S8: Description of the Cluster gene regions of UTNGt2 detected showing similarity on the MIBig database; Supplementary Figure S1: Phylogenetic tree based on the multiple alignment result of the target and selected 16S gene sequences to the gcType 16S rDNA gene database; Supplementary Figure S2: Genes annotated with the CARD protein ID and grouped by Drug Class (B), Resistance Mechanism (B), and AMR gene family (C).
