# Supplementary material for: Genome Characterization of Lactiplantibacillus plantarum Strain UTNGt2 Originated from Theobroma grandiflorum (White Cacao) of Ecuadorian Amazon: Antimicrobial Peptides from Safety to Potential Applications
Source: Antibiotics (Basel). 2021 Apr 3;10(4):383. doi: 10.3390/antibiotics10040383 (PMC8066128; doi:10.3390/antibiotics10040383)
Supplement: Supplementary file 1 [file antibiotics-10-00383-s001.pdf]

antibiotics

MDPI

Article

Genome Characterization of *Lactiplantibacillus plantarum* Strain UTNGt2 Originated from *Theobroma grandiflorum* (White Cacao) of Ecuadorian Amazon: Antimicrobial Peptides from Safety to Potential Applications

Gabriela N. Tenea * and Clara Ortega

Table S1. Assembly summary results.

Sample	Contigs	Total contig bases	N50	Max length	Min length	Mean length
UTNGt2	31	3,264,448	186,152	307,684	1,169	105,304

Contigs: The number of contigs assembled; Total bases of contigs: The total length of contigs; N50: 50% of all contig bases come from contigs longer than this value; Max length: The length of the longest contig; Min length: The length of the shortest contig; Mean length: The average length of contigs assembled.

Table S2. Mapping overall results.

Library name	Total reads	Mapped reads	Coverage (%)	Depth	Ins.size (Std.)
UTNGt2	5,258,542	5,258,086 (99.99%)	100.00	160.15	454.46 (93.64)

Legend: Library name: Sample's library name; Total reads: Total number of reads; Mapped reads: Total number of mapped reads; Coverage (%): The percentage of mapped sited (>= 1x); Depth: Average mapping depth; Ins.size (Std.): The length between adapters and standard deviation of predicted length.

Table S3. BUSCO analysis results.Used Lineage: bacteria_odb9 (number of species: 3663, number of BUSCOs: 148).

Status	# of BUSCOs	Percentage (%)
Complete BUSCOs (C)		
Complete and single-copy BUSCOs (S)	142	95.95
Complete and duplicated BUSCOs (D)	2	1.35
Fragmented BUSCOs (F)	1	0.68
Missing BUSCOs (M)	3	2.03
Total BUSCO groups searched	148	100

Status: A quantitative assessment list of the completeness in terms of expected gene content. The following two conditions were used to create a status: a. Expected range of scores; b. Expected range of length alignments. If both conditions are met, it is classified as Complete (These complete busco matches are either single-copy or duplicated). If length alignments is not met, it is classified

as Fragmented. If both conditions are not met, it is classified as Missing. # of BUSCOs: Identified count in sample; Percentage: Identified percentage in sample.

Table 4. List of reference ID genomes, species, and lineage used for phylogenetic analysis retrieved from the gyType database.

Reference ID	query1/ Gt2	Species	Lineage
GCA_0000143745.1	0.0114/98.97/99.01/99.07/99.07	*Lactobacillus plantarum*	k__Bacteria l p__Firmicutes l c__Bacilli l o__Lactobacillales l f__Lactobacillaceae l g__Lactobacillus l s__Lactobacillus plantarum
GCA_0030641165.1	0.0342/95.08/95.68/95.48/95.49	*Lactobacillus plantarum subsp. argentoratensis*	k__Bacteria l p__Firmicutes l c__Bacilli l o__Lactobacillales l f__Lactobacillaceae l g__Lactobacillus l s__Lactobacillus plantarum
GCA_0030641145.1	0.0883/85.70/87.70/86.10/86.28	*Lactobacillus paraplantarum*	k__Bacteria l p__Firmicutes l c__Bacilli l o__Lactobacillales l f__Lactobacillaceae l g__Lactobacillus l s__Lactobacillus paraplantarum
GCA_0030641185.1	0.1519/79.24/82.55/79.69/79.98	*Lactobacillus pentosus*	k__Bacteria l p__Firmicutes l c__Bacilli l o__Lactobacillales l f__Lactobacillaceae l g__Lactobacillus l s__Lactobacillus pentosus
GCA_0050405405.1	0.1823/74.22/80.23/74.95/75.10	*Lactobacillus plajomi*	k__Bacteria l p__Firmicutes l c__Bacilli l o__Lactobacillales l f__Lactobacillaceae l g__Lactobacillus l s__Lactobacillus plajomi
GCA_0010039045.1	0.1868/76.72/80.49/77.19/77.41	*Lactobacillus herbarum*	k__Bacteria l p__Firmicutes l c__Bacilli l o__Lactobacillales l f__Lactobacillaceae l g__Lactobacillus l s__Lactobacillus herbarum
GCA_004354495.1	0.2301/67.00/#/67.80/68.71	*Pediococcus pentosaceus*	k__Bacteria l p__Firmicutes l c__Bacilli l o__Lactobacillales l f__Lactobacillaceae l g__Pediococcus l s__Pediococcus pentosaceus
GCA_000769695.1	0.2438/65.33/#/65.84/68.11	*Oenococcus alcoholitolerans*	k__Bacteria l p__Firmicutes l c__Bacilli l o__Lactobacillales l f__Leuconostocaceae l g__Oenococcus l s__Oenococcus alcoholitolerans
GCA_000092505.1	0.2438/64.27/#/64.81/66.30	*Leuconostoc kimchii*	k__Bacteria l p__Firmicutes l c__Bacilli l o__Lactobacillales l f__Leuconostocaceae l g__Leuconostoc l s__Leuconostoc kimchii
GCA_000014445.1	0.2438/64.40/#/65.04/66.41	*Leuconostoc mesenteroides*	k__Bacteria l p__Firmicutes l c__Bacilli l o__Lactobacillales l f__Leuconostocaceae l g__Leuconostoc l s__Leuconostoc mesenteroides
GCA_003363255.1	0.2438/64.65/#/65.65/66.69	*Weissella thailandensis*	k__Bacteria l p__Firmicutes l c__Bacilli l o__Lactobacillales l f__Leuconostocaceae l g__Weissella l s__Weissella thailandensis
GCA_012396435.1	0.2438/64.66/#/65.78/67.26	*Weissella hellenica*	k__Bacteria l p__Firmicutes l c__Bacilli l o__Lactobacillales l f__Leuconostocaceae l g__Weissella l s__Weissella hellenica
GCA_900111205.1	0.2438/66.87/#/67.73/68.55	*Pediococcus ethanolidurans*	k__Bacteria l p__Firmicutes l c__Bacilli l o__Lactobacillales l f__Lactobacillaceae l g__Pediococcus l s__Pediococcus ethanolidurans
GCA_000160595.1	0.2438/64.73/#/65.56/67.56	*Leuconostoc mesenteroides subsp. cremoris*	k__Bacteria l p__Firmicutes l c__Bacilli l o__Lactobacillales l f__Leuconostocaceae l g__Leuconostoc l s__Leuconostoc mesenteroides
GCA_001932615.1	0.2438/65.04/#/65.97/67.41	*Weissella jogaejeotgali*	k__Bacteria l p__Firmicutes l c__Bacilli l o__Lactobacillales l f__Leuconostocaceae l g__Weissella l s__Weissella jogaejeotgali
GCA_005405345.1	0.2630/64.94/#/65.65/66.40	*Enterococcus viikkiensis*	k__Bacteria l p__Firmicutes l c__Bacilli l o__Lactobacillales l f__Enterococcaceae l g__Enterococcus l s__Enterococcus viikkiensis
GCA_008086625.1	0.2630/60.97/#/61.64/61.91	*Acetobacterium bakii*	k__Bacteria l p__Firmicutes l c__Clostridia l o__Clostridiales l f__Eubacteriaceae l g__Acetobacterium l s__Acetobacterium bakii
GCM10008912	0.2630/66.95/#/67.91/68.76	*Pediococcus parvulus*	k__Bacteria l p__Firmicutes l c__Bacilli l o__Lactobacillales l f__Lactobacillaceae l g__Pediococcus l s__Pediococcus parvulus
GCA_000577815.1	0.2630/60.42/#/61.42/62.58	*Clostridium saudiense*	k__Bacteria l p__Firmicutes l c__Clostridia l o__Clostridiales l f__Clostridiaceae l g__Clostridium l s__Clostridium saudiense
GCA_000260375.1	0.2630/65.30/#/65.40/67.13	*Leuconostoc carnosum*	k__Bacteria l p__Firmicutes l c__Bacilli l o__Lactobacillales l f__Leuconostocaceae l g__Leuconostoc l s__Leuconostoc carnosum
GCA_002982135.1	0.2630/67.26/#/68.08/69.18	*Pediococcus inopinatus*	k__Bacteria l p__Firmicutes l c__Bacilli l o__Lactobacillales l f__Lactobacillaceae l g__Pediococcus l s__Pediococcus inopinatus
GCA_001437255.1	0.2630/66.72/#/67.53/68.57	*Pediococcus damnosus*	k__Bacteria l p__Firmicutes l c__Bacilli l o__Lactobacillales l f__Lactobacillaceae l g__Pediococcus l s__Pediococcus damnosus
GCA_000373965.1	0.2960/59.41/#/60.76/60.23	*Uliginosibacterium gangwonense*	k__Bacteria l p__Proteobacteria l c__Betaproteobacteria l o__Rhodocyclales l f__Zoogloeaceae l g__Uliginosibacterium l s__Uliginosibacterium gangwonense

GCA_000 012545.1	0.2960/60.02/#/ 60.57/64.33	*Methanosphaera stadtmanae*	k__Archaea|p__Euryarchaeota|c__Methanobacteria|o__Methanobacteriales|f__Methanobacteriaceae|g__Methanosphaera|s__Methanosphaera stadtmanae
GCA_000 320405.1	0.2960/60.48/#/ 61.63/62.32	*Clostridium celatum*	k__Bacteria|p__Firmicutes|c__Clostridia|o__Clostridiales|f__Clostridiaceae|g__Clostridium|s__Clostridium celatum
GCA_000 258405.1	0.2960/61.15/#/ 61.77/63.54	*Ignavibacterium album*	k__Bacteria|p__Ignavibacteriae|c__Ignavibacteria|o__Ignavibacteriales|f__Ignavibacteriaceae|g__Ignavibacterium|s__Ignavibacterium album
GCA_000 015285.1	0.2960/61.20/#/ 61.75/61.94	*Psychromonas ingrahamii*	k__Bacteria|p__Proteobacteria|c__Gammaproteobacteria|o__Alteromonadales|f__Psychromonadaceae|g__Psychromonas|s__Psychromonas ingrahamii
GCA_000 166735.2	0.2960/64.21/#/ 65.08/66.82	*Leuconostoc inhae*	k__Bacteria|p__Firmicutes|c__Bacilli|o__Lactobacillales|f__Leuconostocaceae|g__Leuconostoc|s__Leuconostoc inhae
GCA_000 367405.1	0.2960/61.74/#/ 62.87/64.78	*Rickettsia prowazekii*	k__Bacteria|p__Proteobacteria|c__Alphaproteobacteria|o__Rickettsiales|f__Rickettsiaceae|g__Rickettsia|s__Rickettsia prowazekii
GCA_000 297255.1	0.2960/61.00/#/ 61.63/63.63	*Nitrolancea hollandica*	k__Bacteria|p__Chloroflexi|c__Thermomicrobia|o__Sphaerobacterales|f__Sphaerobacteraceae|g__Nitrolancea|s__Nitrolancea hollandica
GCA_000 378365.1	0.2960/61.23/#/ 62.32/63.75	*Megamonas rupellensis*	k__Bacteria|p__Firmicutes|c__Negativicutes|o__Selenomonadales|f__Selenomonadaceae|g__Megamonas|s__Megamonas rupellensis
GCA_000 173355.1	0.2960/60.89/#/ 61.61/62.61	*Anaerococcus hydrogenalis*	k__Bacteria|p__Firmicutes|c__Tissierellia|o__Tissierellales|f__Peptoniphilaceae|g__Anaerococcus|s__Anaerococcus hydrogenalis
GCA_000 378225.1	0.2960/61.18/#/ 62.19/64.02	*Methylophilus methylotrophus*	k__Bacteria|p__Proteobacteria|c__Betaproteobacteria|o__Nitrosomonadales|f__Methylophilaceae|g__Methylophilus|s__Methylophilus methylotrophus
GCA_000 153185.1	0.2960/61.16/#/ 61.76/63.31	*Reinekea blandensis*	k__Bacteria|p__Proteobacteria|c__Gammaproteobacteria|o__Oceanospirillales|f__Saccharospirillaceae|g__Reinekea|s__Reinekea blandensis
GCA_000 008045.1	0.2960/62.74/#/ 63.34/63.83	*Rickettsia typhi*	k__Bacteria|p__Proteobacteria|c__Alphaproteobacteria|o__Rickettsiales|f__Rickettsiaceae|g__Rickettsia|s__Rickettsia typhi
GCA_000 257395.1	0.2960/62.04/#/ 63.18/64.64	*Diplorickettsia massiliensis*	k__Bacteria|p__Proteobacteria|c__Gammaproteobacteria|o__Legionellales|f__Coxiellaceae|g__Diplorickettsia|s__Diplorickettsia massiliensis
GCA_000 376985.1	0.2960/63.94/#/ 64.47/65.11	*Streptococcus henryi*	k__Bacteria|p__Firmicutes|c__Bacilli|o__Lactobacillales|f__Streptococcaceae|g__Streptococcus|s__Streptococcus henryi
GCA_000 300235.2	0.2960/61.40/#/ 62.05/63.59	*Thalassospira xiamenensis*	k__Bacteria|p__Proteobacteria|c__Alphaproteobacteria|o__Rhodospirillales|f__Rhodospirillaceae|g__Thalassospira|s__Thalassospira xiamenensis
GCA_000 299895.1	0.2960/61.44/#/# /63.61	*Idiomarina xiamenensis*	k__Bacteria|p__Proteobacteria|c__Gammaproteobacteria|o__Alteromonadales|f__Idiomarinaceae|g__Idiomarina|s__Idiomarina xiamenensis
GCA_000 190595.1	0.2960/60.49/#/ 61.66/62.73	*Cellulophaga lytica*	k__Bacteria|p__Bacteroidetes|c__Flavobacteriia|o__Flavobacteriales|f__Flavobacteriaceae|g__Cellulophaga|s__Cellulophaga lytica

Table S5. EggNOG category distribution result.

Eggnog	Description	Count	Ratio (%)
J	Translation, ribosomal structure and biogenesis	150	5.0573
A	RNA processing and modification	0	0
K	Transcription	251	8.4626
L	Replication, recombination and repair	139	4.6864
B	Chromatin structure and dynamics	0	0
D	Cell cycle control, cell division, chromosome partitioning	25	0.8429
Y	Nuclear structure	0	0
V	Defense mechanisms	70	2.3601
T	Signal transduction mechanisms	73	2.4612
M	Cell wall/membrane/envelope biogenesis	166	5.5968
N	Cell motility	3	0.1011
Z	Cytoskeleton	0	0
W	Extracellular structures	0	0
U	Intracellular trafficking, secretion, and vesicular transport	23	0.7755
O	Posttranslational modification, protein turnover, chaperones	63	2.1241
C	Energy production and conversion	110	3.7087

G	Carbohydrate transport and metabolism	279	9.4066
E	Amino acid transport and metabolism	201	6.7768
F	Nucleotide transport and metabolism	86	2.8995
H	Coenzyme transport and metabolism	72	2.4275
I	Lipid transport and metabolism	56	1.8881
P	Inorganic ion transport and metabolism	137	4.619
Q	Secondary metabolites biosynthesis, transport and catabolism	17	0.5732
R	General function prediction only	300	10.1146
S	Function unknown	745	25.118
**Total**	**-**	**2966**	**100**

count: number of genes; ratio (%): % of genes

Table S6. Antibiotic susceptibility of the UTNGt2 strain.

**Antimicrobial E-test strip**	**MIC (mg/L)**	**Susceptibility***	**EFSA cut-off values (mg/L) *Lactobacillus* obligate Heterofermentative [EFSA, 2012]**
Amoxicillin	0.5	S	4
Amoxicillin: clavulanic acid	0.016	S	4
Ampicillin	0.5	S	2
Cefotaxime	0.002	S	16
Erythromycin	2	S	1
Gentamycin	16	S	16
Penicillin	0.25	S	0.25
Tetracycline	32	S	32**

*The microbiological breakpoints reported by the FEEDAP were used to categorize lactobacilli as susceptible or resistant. The strains showing a MIC higher than the EFSA breakpoint were considered resistant. Susceptible (S): a bacterial strain is defined as susceptible when it is inhibited at a concentration of a specific antimicrobial equal to or lower than the established cut-off value (S ≤ x mg / L). Resistant (R): a bacterial strain is defined as resistant when it is not inhibited at a concentration of a specific antimicrobial above the established cut-off value (R> x mg / L). ** MIC reference for *L. plantarum.*

Table S7. Summary of Prophage regions identified on the UTNGt2 genome using the PHASTER webserver.

Contig	Region	Region length	Completeness	Score	# Total proteins	Region position	Most common Phage	GC%
2	1	3.3 kb	incomplete	20	6	214956-218324	PHAGE_Xantho_Carpasina_NC_047962(1)	49.04
3	1	6.2 kb	incomplete	10	6	156134-162384	PHAGE_Bacill_G_NC_023719(3)	47.85
5	1	9.4 kb	incomplete	10	11	121969-131437	PHAGE_Vibrio_YC_NC_048709(1)	44.83
6	3	39.9 kb	intact	150	50	16543-56481	PHAGE_Lactob_Sha1_NC_019489(27)	40.79

Legend: region: the number assigned to the region; region length: the length of the sequence in that region; completeness: a prediction of whether the region contains a intact or incomplete prophage based on the criteria: intact (score > 90%); questionable (score 70-90); incomplete (score < 70%); score: the score of the region based on the mentioned criteria; # total proteins: the number of ORFs present in the region; Region position: the start and end positions of the region on the bacterial chromosome; Most common phage: the phages (s) with the highest number of proteins most similar to those in the region; GC%: the percentage of GC nucleotides of the region.

Table S8. Description of the Cluster gene regions of UTNGt2 detected showing similarity on the MIBig database.

Reference ID	Similarity score	Type	Compound(s)	Organism
BGC0000617.1	0.22	RiPP	coagulin	*Bacillus coagulans*
BGC0001407.1	0.16	RiPP	bicereucin	*Bacillus cereus* SJ1
BGC0001930.1	0.16	RiPP	pallidocin	*Aeribacillus pallidus*
BGC0001862.1	0.16	RiPP	geocillicin	*Aeribacillus pallidus*
BGC0000558.1	0.16	RiPP	sublancin 168	*Bacillus subtilis subsp. subtilis str.* 168
BGC0001863.1	0.15	RiPP, Terpene	bacillicn CER074	*Bacillus mycoides*
BGC0001861.1	0.15	RiPP	bacillicin BAG2O	*Bacillus cereus* BAG2O-1
BGC0000484.1	0.15	RiPP	glycocin F	*Lactobacillus plantarum*
BGC0000500.1	0.14	RiPP	carnolysin A1, carnolysin A2	*Carnobacterium maltaromaticum*
BGC0000504.1	0.14	RiPP	cytolysin ClyLl, cytolysin ClyLs	Plasmid pAD1
BGC0000647.1	0.45	Terpene	carotenoid	*Rhodobacter sphaeroides*
BGC0000648.1	0.23	Terpene	carotenoid	*Myxococcus xanthus*
BGC0000656.1	0.23	Terpene	zeaxanthin	*Xanthobacter autotrophicus* Py2
BGC0000637.1	0.23	Terpene	carotenoid	*Corynebacterium glutamicum*
BGC0000645.1	0.21	Terpene	carotenoid	*Halobacillus halophilus* DSM 2266
BGC0001227.1	0.21	Terpene	isorenieratene	*Streptomyces collinus* Tu 365
BGC0000633.1	0.21	Terpene	carotenoid	*Streptomyces avermitilis*
BGC0000640.1	0.2	Terpene	carotenoid	*Enterobacteriaceae bacterium* DC404
BGC0000636.1	0.2	Terpene	carotenoid	*Brevibacterium linens*

BGC0000630.1	0.2	Terpene	(2R,3S,3'S)-2-hydroxyastaxanthin	*Paracoccus haeundaensis*
BGC0000286.1	0.19	Polyketide	viguiepinol	*Streptomyces sp.* KO-3988
BGC0000554.1	0.16	RiPP	SRO15-3108	*Streptomyces filamentosus* NRRL 15998
BGC0001551.1	0.16	RiPP	citrulassin E	*Streptomyces glaucescens*
BGC0000386.1	0.16	NRP	malleobactin A, malleobactin B, malleobactin C, malleobactin D	*Burkholderia thailandensis* E264
BGC0000551.1	0.16	RiPP	SapB	*Streptomyces coelicolor* A3(2)
BGC0000205.1	0.16	Polyketide	bryostatin	*Candidatus Endobugula sertula*
BGC0000489.1	0.16	RiPP	enterocin AS-48	*Enterococcus faecalis*
BGC0000504.1	0.16	RiPP	cytolysin ClyLl, cytolysin ClyLs	Plasmid pAD1
BGC0000500.1	0.16	RiPP	carnolysin A1, carnolysin A2	*Carnobacterium maltaromaticum*
BGC0000490.1	0.16	RiPP	garvicin ML	*Lactococcus garvieae* DCC43

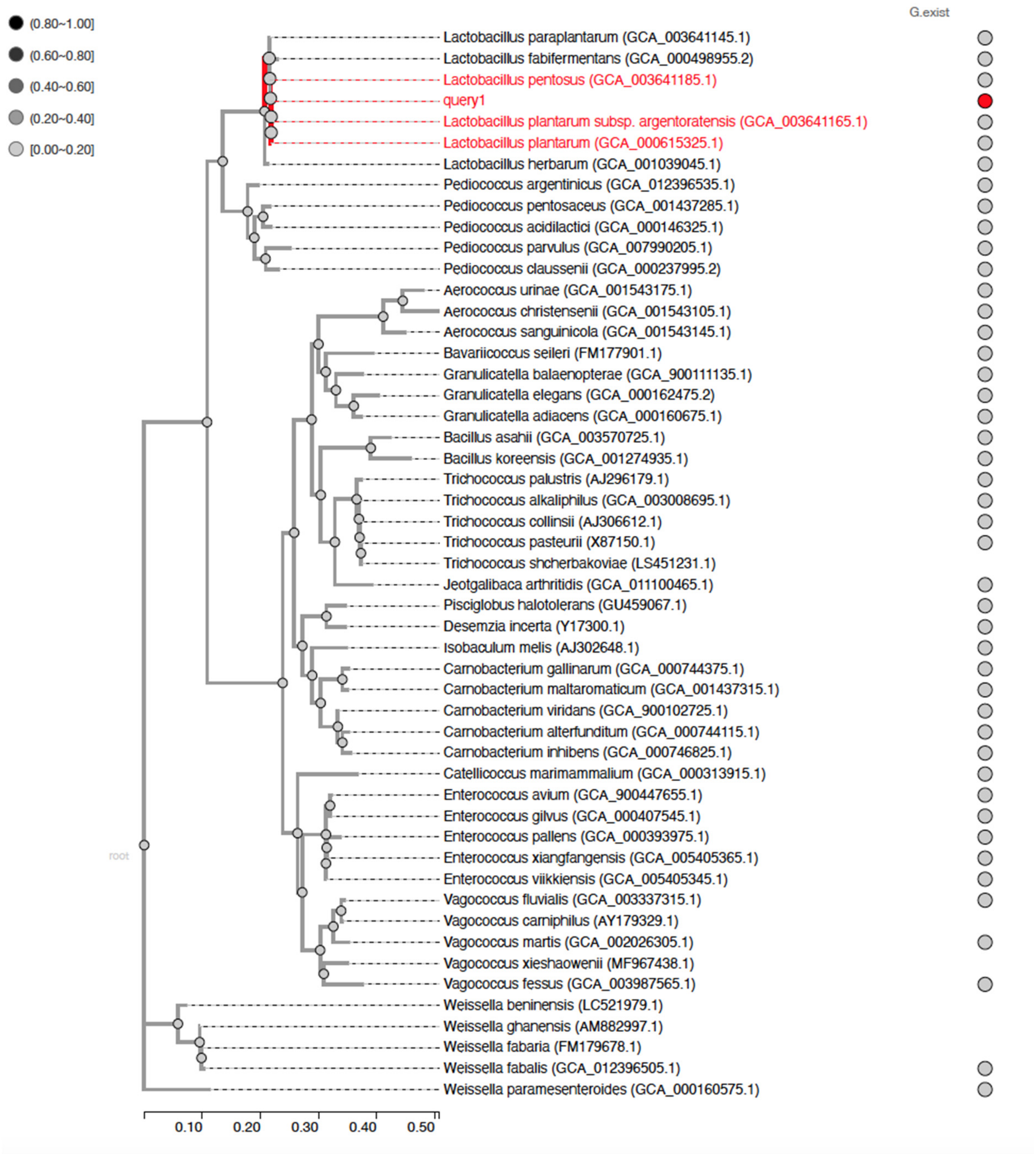

Figure S1. Phylogenetic tree based on the multiple alignment result of the target and selected 16S gene sequences to the gcType 16S rDNA gene database. Query 1: UTNGt2.

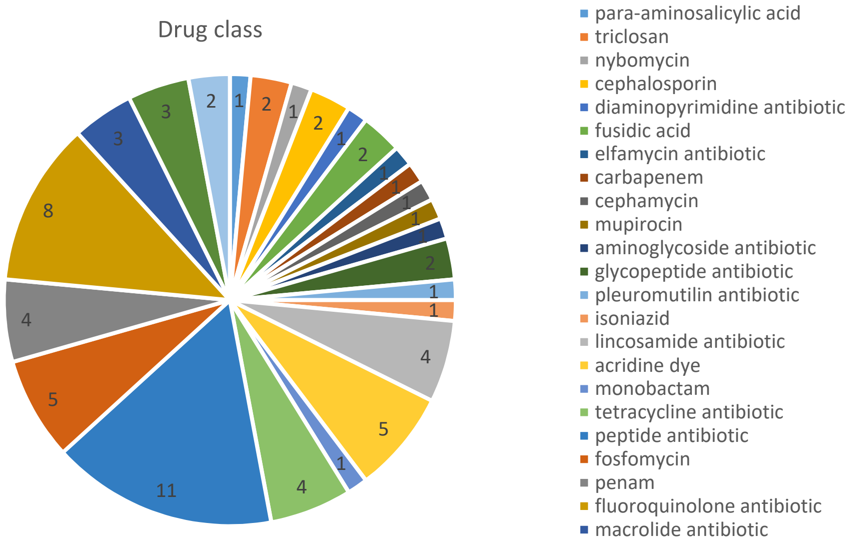

A.

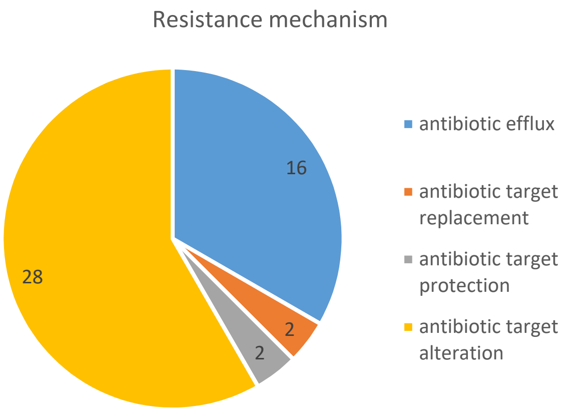

B.

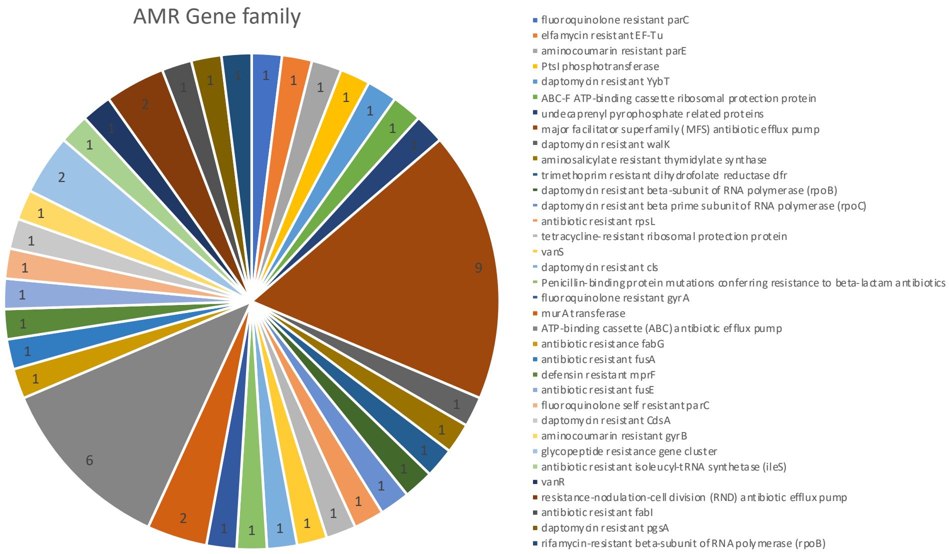

C.

Figure S2. Genes annotated with the CARD protein ID and grouped by Drug Class (B), Resistance Mechanism (B) and AMR gene family (C). The number of genes in each category is shown.
